# A Comparative Study of Group Behavioral Activation and Cognitive Therapy in Reducing Subsyndromal Anxiety and Depressive Symptoms

**Published:** 2015-04

**Authors:** Mehdi Soleimani, Parvaneh Mohammadkhani, Behroz Dolatshahi, Hamid Alizadeh, Karenleigh A. Overmann, Frederick L. Coolidge

**Affiliations:** 1 Department of Psychiatry, Tehran University of Medical Sciences, Tehran, Iran; 2 University of Social Welfare and Rehabilitation Sciences, Tehran, Iran; 3 Allameh Tabataba’i University, Tehran, Iran; 4 Department of Psychology, University of Colorado, Colorado Springs, USA

**Keywords:** *Behavioral Activation Therapy*, *Cognitive Therapy*, *Subsyndromal Anxiety and Depressive Symptoms*, *Functional Impairment*

## Abstract

**Objective**: This study compared the effectiveness of two group treatments, behavioral activation (BA) and cognitive therapy (CT), in reducing subsyndromal anxiety and depressive symptoms in a sample of Iranian university students.

**Method:** Twenty-seven Iranian university students who scored 18 or higher on the depression subscale and 16 or higher on the anxiety subscale of the Depression, Anxiety, and Stress Scale (DASS-42) were randomly assigned into treatment groups. One group received 8 sessions of BA (n = 14), and the other received 8 sessions of group CT (n = 13).

**Result:** Analysis of covariance revealed that the BA group had a significantly greater reduction in depressive symptoms than the CT group. However, there were no significant differences between the two groups in the levels of anxiety, stress symptoms or functional impairment after treatment.

**Conclusion:** This study found evidence for the effectiveness of BA in reducing anxiety, depressive and stress symptoms and functional impairment compared to CT. BA was more effective than CT in improving depressive symptoms and was as effective as CT in decreasing anxiety, stress and functional impairment. BA is also a cost-effective intervention, particularly in group formats**.**

According to the World Health Organization (WHO), anxiety and depressive disorders are the most common and prevalent mental disorders, with anxiety disorders as the most prevalent mental disorder (1, 2). The rate of comorbidity between anxiety and depressive disorders is also significant (3, 4). For example, the average rate of comorbidity between major depressive disorder and anxiety disorders exceeds 50% (5).

Subsyndromal anxiety and depression, though clinically failing to meet formal diagnostic criteria, has a significant potential to impair day-to-day functioning (6). Despite the fact that subsyndromal anxiety and depressive symptoms have a tendency to become chronic, they often receive little clinical attention, prompting the need to develop effective treatment strategies (7–9). Moreover, patients with subsyndromal anxiety and depressive symptoms are at greater risk than the general population for developing clinical anxiety and depressive disorders when faced with psycho-social stressors (10).

Epidemiological studies have found an elevated rate of anxiety and depressive symptoms in university students in Iran (11–13), with symptomatic severity interfering with students’ ability to function in the educational setting (14).

Cognitive therapy (CT) and behavior therapy have demonstrated effectiveness in treating anxiety and depressive disorders (15–17). There is evidence suggesting that current psychotherapy models can reduce subsyndromal symptoms (18). Moreover, a new trend in the psychotherapy research, the transdiagnostic (unified) approach (19, 20), emphasizes the common etiology among emotional disorders, providing support for taking a dimensional (rather than categorical) approach to comorbid mental disorders (21). The transdiagnostic approach has several important features: It is easy to train clinicians to administer the interventions, it has the capacity to be implemented in a group setting, it can be applied to comorbid anxiety and depression, and it has demonstrated effectiveness in preventing relapse in recovery from emotional disorders (22, 23). Furthermore, the transdiagnostic approach does not require formal clinical diagnosis of a psychiatric disorder (22).

Outcome studies have indicated the efficacy of CT for a variety of mental disorders, including major depressive disorder, generalized anxiety disorder, panic disorder and social anxiety disorder (16, 24). CT is also effective in the treatment of mixed anxiety–depressive disorders (25) and in heterogeneous anxiety disorders in group format (26). Furthermore, preventive studies with at-risk populations indicate the potential effectiveness of CT-based programs (27, 28). Alternatively, medication (Fluvoxamine and Sertraline) has also demonstrated efficacy for treating mixed anxiety-depressive disorder in short-term periods (8 weeks) of treatment (29, 30).

Behavioral activation (BA) is a behaviorally-oriented psychotherapy that helps clients identify and modify inactivity and avoidance patterns (31). It was originally developed to treat depression (32) and has shown efficacy for treating this disorder (16, 33). BA has also been used to treat other disorders that are considered as emotion dysregulation, including anxiety disorders (34).

Avoidant behavior plays a key role in fostering and maintaining anxiety disorders and depression. For example, depressed patients are frequently involved in avoidant behaviors like complaining, which means they often lose any chance of gaining positive reinforcement (35). These insights into the connection between avoidant behavior and anxiety and depressive disorders inform both behavior therapy (which helps patients regulate their emotions by changing their behavior (36)) and exposure and response prevention (ERP, an empirically validated psychotherapy for anxiety disorders (16, 37)).

Because BA targets avoidant behaviors, the treatment is appropriate for managing comorbid anxiety and depression (34, 38). There is some early evidence showing that BA can decrease symptoms of posttraumatic stress disorder and comorbid major depressive disorder (39, 40). Pilot studies have shown that BA has positive effects in decreasing anxiety symptoms in chronic anxiety and coexistent depressive and anxiety symptoms (41, 42). Chu and colleagues (43) also found that group BA therapy had clinical benefits for anxious and depressed young adolescents.

Group and short-term modalities yield several benefits, including cost-effectiveness and an ability to cover more patients than is possible with individual therapies or long-term treatments (44). Group and individual therapies have demonstrated similar effectiveness in treatment outcomes (45), and the reduced complexity of BA makes it consistent with the transdiagnostic approach (33, 46).

It is important to note that there have been a number of recent empirical studies that support the use of BA and CT in a variety of settings and populations (e.g., see 47–56). Thus, the purpose of this study was to compare the effectiveness of group BA treatment and group CT to reduce subsyndromal anxiety and depressive symptoms, stress symptoms and functional impairment in Iranian university students.

## Material and Methods

*Participants*

Participants were Iranian university students (n = 32) recruited through advertisements in several universities in Tehran. This sample size was determined on the basis of Cohen’s table (57) for a power of .08, alpha of .05, and an effect size of .80. The sample size exceeded the recommendation of Butler and colleagues (24), who suggest that a small number of participants (n = 9) is acceptable in studies of cognitive therapies for depression and anxiety. There were a total of 20 female participants, with 11 being assigned to the BA condition; of the seven male participants, three were assigned to the BA condition. The average age of the participants was 22 years for the BA condition and 23 years for the CT condition. Of the 14 participants assigned to the BA condition, only one was married, while in the 13 participants in the CT condition, two were married. These demographics are presented in Table 1.

Criteria for participant inclusion in the study were the existence of subsyndromal anxiety and depressive symptoms as measured by a score of 18 or higher on the depression subscale and 16 or higher on the anxiety subscale of the Depression, Anxiety and Stress Scale (DASS-42). This cutoff was selected to reflect the criteria used by Sahebi and colleagues (58) in their study on validating DASS-21 findings in a sample of an Iranian population, in which a score of 9 on the depression subscale and a score of 8 on the anxiety subscale of the DASS-21 indicated inclusion in the 75th percentile and a mild level of symptom severity. The items of the DASS-21 were selected to reflect all of the subscales, and the DASS-21subscale scores were multiplied by 2 to create the DASS-42 scales. Exclusion criteria were the existence of current Axis I disorders, including anxiety, depression, psychotic, somatoform or substance abuse, assessed by diagnostic interviews based on the Anxiety Disorders Interview Schedule for DSM-IV (ADIS-IV) (59) and simultaneously being on prescribed medication or in psychotherapy. All participants agreed to participate in the study and provided written informed consent.

The dropout criterion was three absences in eight sessions, consistent with the recommendation of Gollan and colleagues (60). Two participants dropped out of the BA condition, and three dropped out of the CT condition, reducing the total number of participants to 27. One participant left after two CT sessions due to dissatisfaction with the intervention relative to perceived needs. Another participant in the CT condition was unable to continue with the intervention following the death of a family member. Three other participants dropped out due to schedule conflicts.

*Materials and Procedure*

This study compared two groups of BA as the experimental condition and CT as the comparison condition. Participants were randomly assigned into one of the groups. CT was considered the comparison condition because of its demonstrated efficacy in treating depressive and anxiety disorders (15, 17) and its widespread use as a psychotherapeutic treatment (62). CT is also the most well-known and widely used psychotherapy in Iran, and its individual and group formats have been subjected to numerous investigations in treating Iranian patients (63–66). Thus, the CT condition was considered to be the standard of comparison for the treatments in this study. CT presented the additional benefits of enabling control of non-specific therapeutic factors (e.g., contacting the therapist and attending treatment sessions) and provided an ethical treatment alternative for the comparison group.

Recruitment advertisements were placed at several Iranian universities. Volunteers were individually evaluated onsite to confirm their eligibility for the study and obtain informed consent. For determining eligibility, a clinical interview using ADIS-IV and DASS-42 was conducted for each participant. To measure functional impairment, the participants were asked to complete the Work and Social Adjustment Scale (WSAS) (67). Participants were then randomly assigned into either the BA or the CT treatment groups. Each treatment group consisted of 16 participants who received eight sessions of group intervention led by a trained therapist. To meet the recommended participant-to-therapist ratio (68), each group was further divided into two 8-member groups. One therapist conducted both BA subgroups, while the CT sessions had separate therapists for each subgroup. To ensure therapist compliance with the CT manual, peer supervision was used. Each CT therapist attended three sessions (sessions 1, 4, and 6) conducted by the other therapist as a co-therapist to monitor adherence to the manual. After each session, the two therapists discussed the session’s process and content and provided recommendations to improve compliance with the manual. Finally, all participants completed the DASS-42 and the WSAS at the post-treatment assessment.

*Treatment and Therapists*

BA treatment followed the guidelines of Gollan and Martell and colleagues (31, 60, 61) and consisted of eight group sessions. Each session had an agenda for the therapist to follow (outlined in Table 2) and lasted for 90 minutes. The primary goal of BA is to teach the participants to identify the patterns of behavior (especially avoidance behaviors) associated with anxiety and depressive symptoms, and to change the behaviors in ways that improve emotional regulation. Participants were encouraged to stop avoiding situations that led to feelings of anxiety and depression in favor of making changes that could positively alter emotional states. Participants were also encouraged to increase their level of life activity to increase their possibilities of feeling enjoyment and accomplishment.

CT followed the published guidelines (68). As in the BA condition, CT consisted of eight group sessions, each of which was implemented by a therapist according to a pre-established agenda (Table 2) and lasted for 90 minutes.

Participants in the CT condition were asked to identify relations between their thoughts and emotions, automatic thoughts and cognitive distortions. They were then encouraged to challenge them with cognitive techniques.

The two CT therapists were both PhD students in a clinical psychology program with at least 2 years of experience in the psychotherapy of anxiety and depressive disorders obtained in university counseling centers. They were also trained in cognitive behavioral therapy for the treatment of anxiety and depressive disorders. Prior to the study, they reviewed treatment manuals, and during the study, they received copies of session agendas.

*Measures*

*Anxiety, Depressive and Stress Symptoms*: The Depression, Anxiety and Stress Scale (DASS) (69) was used to assess anxiety, depressive and stress symptoms. The DASS is a 42-item self-report questionnaire developed to measure three relevant emotions (depression, anxiety and stress). This questionnaire has a 21-item form. The measure is a valid and reliable instrument for the Iranian population, as assessed by Sahebi and colleagues (58) for the DASS-21 and by Bakhshipour and Dejkam (70) for the DASS-42. Sahebi and colleagues also established the construct validity of the DASS-21 in Iranian samples through factor analysis, and they found the internal reliability (Cronbach’s α) of the DASS-21 in an adult Iranian sample of α = .77 for the depression subscale, α = .79 for anxiety subscale and α = .78 for stress subscale. Moreover, Bakhshipour and Dejkam reported α coefficients for the DASS-42 subscales, depression, anxiety and stress, respectively, of α = .97, α = .92, and α = .94. The construct validity of the DASS-42 was also established through factor analysis (70).

*Diagnostic Interview:* The Anxiety Disorders Interview Schedule for DSM-IV (ADIS-IV) (59) was used to determine the diagnostic status of the participants. The ADIS-IV is a reliable structured interview designed to assess current anxiety disorders based on DSM-IV criteria. The ADIS-IV provides the possibility to assess current mood, somatoform and substance use disorders. The interview also screens for conversion and psychotic symptoms. Brown, Di Nardo, Lehman and Campbell (71) reported good reliability for the lifetime version of the ADIS-IV (ADIS-IV-L) based on kappa coefficients ranging from .58 to .81.

*Functional Impairment:* In order to assess functional impairment, the five-item Work and Social Adjustment Scale (WSAS) was used, which has been shown to be a valid and reliable tool to assess anxiety and depressive disorders (67). We evaluated psychometric characteristics of the WSAS in a pilot study with a sample of Iranian university students (N = 67, age mean = 22.8 years, standard deviation of age = 2.3 years). According to this evaluation, there was a positive and significant Pearson correlation between the WSAS and the depression subscale of the DASS-21 and the Anxiety subscale of the DASS-21, respectively, r = .66, p < .001 and r = .66, p < .001. Test–retest reliability in a one week period for the WSAS was r = .69, p < .001 (n = 32).

## Results

Participants who were assigned into the two conditions did not differ in their gender, age or marital status (Table 1).

Analysis of Variance (ANOVA) was used to determine the equivalence of the treatment conditions on outcome measures at the pretest point. 

The results did not indicate a significant difference between BA and CT conditions on outcome measures at the pretest point for depressive symptoms, F(1, 25) = 1.13, p > .05; for anxiety symptoms, F(1, 25) = 0.02, p > .05; for stress symptoms, F(1, 25) = 1.44, p > .05; for functional impairment, F(1, 25) = 0.18, p > .05. These results demonstrated the success of random assignment in producing equivalent groups on these variables at the pretest point.

To analyze the effect of each treatment condition on outcome measures, the paired t test was used. Results are presented in Table 3. All paired t tests were significant at p < .01. These results indicated a significant difference between the pretest and posttest scores of each outcome measure in the BA and CT conditions, meaning that both treatments reduced the severity of depressive, anxiety and stress symptoms, as well as functional impairment.

**Table 1:** Participant Characteristics by Treatment Condition

**Variable**	**Behavioral Activation (n = 14)**			**Cognitive Therapy (n = 13)**			**F(1, 25)**	**χ** ^2^ **(N = 1, 27)**
	**M (SD)**	**n**	**%**	**M (SD)**	**n**	**%**		
Gender Female Male								.31
		11	78.6		9	69.2		
		3	21.4		4	30.8		
Age	22.28 (3.07)			23.46 (3.41)			.89	
Marriage Single Married								.46
		13	92.0		11	84.6		
		1	7.1		2	15.4		

**Table 2:** Session Agenda by Treatment Condition

**Session**	**Condition**	
	**Behavioral Activation** a	**Cognitive Therapy** b
1	Negative emotions: Anxiety and depressive symptoms	The three systems model of human emotion and ABC model
2	BA model of anxiety and depression (the role of negative life events, avoidance from situations and others, and rumination in maintaining negative emotions)	Cognitive theory of anxiety and depression (core beliefs, automatic thoughts, negative cognitive triad, and logical errors)
3	Pleasure-accomplishment rating and developing a pleasure activity chart	Thought injection and vertical arrow procedure
4	ACTION skill	Advanced vertical arrow and categorizing beliefs
5	TRAP and TRAC skill	Changeability of beliefs, objective analysis, and utility analysis
6	Stress, depression and anxiety	Logical analysis
7	Assertiveness	countering
8	Review of program and maintenance plan	Review of program and maintenance plan

Note*:* ABC = activating event, belief and emotional consequences; ACTION = assess, choose, try, integrate, observe result and never give up; TRAP = trigger, response and avoidance pattern; TRAC = trigger, response and alternative coping.

a Adapted from (60).

b Adapted from (68).

**Table 3:** Means, Standard Deviations and ANCOVA Results for the Outcome Measures

**Variable**	**Behavioral Activation (n = 14)**			**Cognitive Therapy (n = 13)**			**F(1,24)**
	**Pretest**	**Posttest**	**Paired ** **t(13)** a	**Pretest**	**Posttest**	**Paired ** **t(12)** a	
	**M (SD)**	**M (SD)**		**M (SD)**	**M (SD)**		
DASS-D	23.71(3.83)	12.36(5.98)	8.19	22.15(3.78)	16.46(4.52)	4.81	7.80^b^
DASS-A	20.93(4.03)	12.57(5.12)	6.97	20.69(3.68)	11.46(4.05)	5.99	.35
DASS-S	20.50(4.52)	13.21(3.26)	6.80	18.54(3.93)	11.77(2.98)	5.94	.48
WSAS	19.93(6.13)	13.14(6.69)	3.48	18.85(7.02)	12.54(5.78)	4.74	.00

Note*: *DASS-D = depression subscale of the DASS; DASS-A = anxiety subscale of the DASS; DASS-S = stress subscale of the DASS; WSAS = functional impairment.

a All paired t values were significant at p < .01.

b p < .05.

To compare the BA and CT conditions on the outcome measures, Analysis of Covariance (ANCOVA) was used with pretest scores as the covariate. The ANCOVA results and the means and standard deviations of the outcome measures at the pretest and posttest points are presented in Table 3. 

The BA condition showed a significantly higher effectiveness relative to the CT condition in treating depressive symptoms, F(1, 24) = 7.80, p < .05, partial η^2^ = .24 (a large effect), but there was no difference between the two treatment conditions in improving anxiety and stress symptoms or functional impairment (p > .05) for anxiety symptoms, partial η^2^= .01; for stress symptoms, partial η^2^= .02; for functional impairment, partial η^2^= .00. The effect size (Cohen’s d) for BA relative to CT was also calculated from the ANCOVA F value (72): For depressive symptoms, d = –0.86; for anxiety symptoms, d = 0.21; for stress symptoms, d = 0.24; for functional impairment, d = 0.01. A negative d score was interpreted as indicating the greater effectiveness of BA relative to CT, and a positive d score as indicating the greater effectiveness of CT relative to BA.

According to the definition of a clinically significant change (73), two treatment conditions were compared to determine whether either one moved participants from the range considered dysfunctional to the range assessed as functional. Based on the normative data on the Iranian population for the DASS provided by Sahebi and colleagues (58), cutoff points between the normal level and the level of mild symptom severity were selected for the three subscales. Where Sahebi and colleagues reported different cutoff points for men and women, the present study established different cutoff points to assess the percentage of participants in each condition indicating a clinically significant change in symptom severity.

For depressive symptoms, 71% of the participants of BA condition (n = 10) were assessed to be in the functional range, compared with 31% of CT participants (n = 4). For anxiety symptoms, 50% of BA participants (n = 7) showed a clinically significant improvement in symptoms, compared to 61% of the CT participants (n = 8). Finally, all participants in both conditions reached the functional range in stress symptoms.

## Discussion

This study compared the effectiveness of BA and CT administered in group settings in university students with subsyndromal anxiety and depressive symptoms. The results revealed that both treatments could decrease the severity of symptoms and functional impairments in participants. However, BA was more effective than CT in reducing depressive symptoms as assessed through both statistical and clinical significance. This finding was consistent with a previous, large sample investigation (33) that found BA was more effective than CT in treating severely depressed patients. The effect size reported by Dimidjian and colleagues (33) for the Beck Depression Inventory (BDI) was 0.87 (a large effect), which was comparable to the effect size found by the present study (d = 0.86). In addition to demonstrating greater effectiveness in managing depressive symptoms relative to CT, BA has several advantages. Jacobson and colleagues (46) demonstrated that the BA component of the full CT protocol can be as effective as the full CT protocol, making it easier and less costly to implement. Furthermore, BA is more easily applied to patients and trained in therapists than CT (31, 33).

BA and CT were similarly effective in reducing anxiety, stress symptoms and functional impairment. The finding of this study revealed that BA effectively reduced anxiety symptoms, which is consistent with previous studies (41–43), and it is noteworthy because currently there is only one technique in the BA manual that directly addresses anxiety issues and prescribes exposure treatment. Although there were no in vivo exposure exercises in BA sessions, therapists encouraged participants to expose and stop avoiding. Of course, the characteristics of the present sample might limit the generalization of these findings.

*Limitations and Future Directions*

This was a pilot study; and as a result, there were several limitations. First, the sample size was not large, possibly limiting the generalizability of the results. Therefore, future studies should consider increasing the sample size to improve generalizability. Second, the CT condition in the present study was a subset rather than the full protocol, and this may limit its effectiveness, which can be increased when implemented with other techniques in the complete CT protocol. Finally, the sample was taken from a specific, highly educated population, and the findings might be limited to this population. Future studies should compare the BA and CT interventions on other populations, including clinical samples. Furthermore, it may be useful to investigate the preventive effect of BA and explore the mechanisms of change in the intervention.

## Conclusions

Participants in the present study had subsyndromal symptoms of anxiety and depression. As noted previously, these symptoms can lead to the significant functional impairments (6, 14). As found by this study, BA is an effective treatment for these symptoms and may be preferable to CT in terms of cost and ease of implementation. On the other hand, medications are often prescribed for these symptoms (29, 30), with CT being the most commonly prescribed form of psychotherapy (62), so patients suffering from these symptoms are likely to receive CT treatment interventions in clinical settings. Thus, this study offers BA as an effective alternative to CT in clinical settings.

The effects of both treatments on anxiety and depressive symptoms indirectly support the notion that a common etiology underlies depressive and anxiety disorders, and it is consistent with the transdiagnostic approach (19, 20) to go beyond the current diagnostic categories and develop simpler intervention.

In summary, this study found positive evidence for the effectiveness of BA in reducing anxiety, depressive and stress symptoms and functional impairment relative to CT. BA was more effective than CT in improving depressive symptoms and was as effective as CT in decreasing anxiety, stress and functional impairment. BA is also a cost-effective intervention, particularly in group formats for clients and managed care.
